# Patient-Reported Experiences With Long-Term Lifestyle Self-Monitoring in Heart Disease: Mixed Methods Study

**DOI:** 10.2196/76978

**Published:** 2025-09-17

**Authors:** Mayra Goevaerts, Nicole Tenbült - Van Limpt, Willem J Kop, Hareld Kemps, Yuan Lu

**Affiliations:** 1 Department of Industrial Design Eindhoven University of Technology Eindhoven The Netherlands; 2 Department of Cardiology Máxima Medisch Centrum Eindhoven The Netherlands; 3 Center of Research on Psychological Disorders and Somatic Diseases Department of Medical and Clinical Psychology Tilburg University Tilburg The Netherlands

**Keywords:** cardiac rehabilitation, self-monitoring, wearable electronic devices, chatbots, behavior change

## Abstract

**Background:**

Lifestyle behaviors strongly predict cardiovascular morbidity and mortality, emphasizing the need for strategies that support sustained lifestyle changes in patients with cardiac disease. Digital health solutions, including wearables, mobile apps, and chatbots, enable self-monitoring of lifestyle behaviors but often face challenges with engagement and usability. While self-monitoring systems can increase awareness and accountability, maintaining user engagement remains crucial for their effectiveness in promoting behavior change and long-term improvements.

**Objective:**

This study evaluated patient experiences with a lifestyle monitoring system combining a web application, health watch, and chatbot. We explored facilitators of and barriers to long-term adherence and assessed the impact of self-monitoring on lifestyle awareness and behavior change in patients with cardiac disease.

**Methods:**

We conducted a mixed methods study with patients who used an eHealth platform for self-monitoring lifestyle behaviors during 1 year following an invasive cardiac procedure. This study included 100 patients (mean age 61.6, SD 10.4 y; n=88, 88% male) comprising both completers (n=57, 57%) and dropouts (n=43, 43%). Patients engaged in quarterly phone interviews and questionnaires and completed an end-of-study questionnaire. Completers participated in a structured evaluation interview; dropouts provided a reason for discontinuation. Quantitative and qualitative data analyses focused on usability, long-term adherence facilitators and barriers, lifestyle awareness, and behavior change.

**Results:**

Patients completed 157 quarterly questionnaires (n=145, 92.4% by completers and n=12, 7.6% by dropouts) and 217 phone interviews (n=171, 78.8% with completers and n=46, 21.2% with dropouts). In total, 77 patients (of whom n=54, 70% were completers and n=23, 30% were dropouts) completed end-of-study questionnaires, and 98% (56/57) of completers participated in the evaluation interviews. Completers reported higher perceptions of the platform’s usefulness, ease of use, and satisfaction (*P*<.001 in all cases) than dropouts. Dropout reasons linked to self-monitoring (34/43, 79%) included high self-report burden and dissatisfaction with the chatbot, poor overall usability experience, health watch technical challenges causing frustration, limited perceived usefulness, mental stress from self-monitoring, and low motivation. Key facilitators of long-term engagement included routine formation, structured reminders, and minimal effort associated with the wearable. Barriers included repetitive chatbot questions (causing cognitive burden) and technical issues with the health watch. Self-monitoring increased lifestyle awareness among completers, particularly regarding physical activity (25/56, 45%) and nutrition (29/56, 52%), with smaller effects for sleep quality (7/56, 13%) and mental stress (1/56, 2%). It facilitated behavior change in physical activity and nutrition (16/56, 29% each) and sleep quality (4/56, 7%) but not in mental stress. Adaptive personalization, mobile accessibility, and real-time feedback could improve adherence.

**Conclusions:**

Fostering routine formation while minimizing patient burden through personalized, flexible, and adaptive features is important for sustained patient engagement in eHealth monitoring systems. Enhancing relevance and usability while reducing complexity and technical barriers can optimize digital health tools and promote lasting behavior change.

**International Registered Report Identifier (IRRID):**

RR2-10.1186/s12872-023-03222-x

## Introduction

### Background

Cardiovascular disease remains a leading cause of morbidity and mortality worldwide [1], constituting a significant burden on health care systems [2] and highlighting the need for effective secondary prevention strategies. While (elective) medical interventions such as coronary artery bypass graft (CABG) surgery; percutaneous coronary intervention (PCI); radiofrequency catheter ablation; and valve surgery, including transcatheter aortic valve implantation, improve short-term outcomes (ie, reduce symptom burden and enhance short-term quality of life) [3-5], long-term patient-reported outcomes rely heavily on patients’ ability to sustain healthy lifestyle behaviors [6-8]. However, maintaining healthy lifestyle behaviors over time remains challenging as many patients struggle with adherence to recommended behaviors such as regular physical activity, balanced nutrition, adequate sleep, and stress management [9]. Therefore, there is a growing interest in strategies that promote sustained lifestyle change after a cardiac intervention.

Digital health solutions such as wearable devices, mobile apps, and automated chatbot systems have emerged as promising tools for supporting patients with cardiac disease in self-monitoring their lifestyle behaviors with the aim of facilitating long-term behavior change beyond traditional cardiac rehabilitation [10]. While these technologies enhance engagement and adherence during the initial recovery period [11], long-term adoption remains a challenge [12]. Many interventions are associated with high dropout rates, yet the factors influencing sustained use over time are not completely understood. While continuous self-monitoring can enhance motivation [13], it may also lead to burden or disengagement, particularly if usability or system design issues are not adequately addressed [14]. A deeper understanding of these dynamics is crucial for optimizing digital interventions to promote long-term adherence [15] and integration into routine clinical care.

To effectively understand and support sustained behavior change, it is essential to examine not only the clinical efficacy of digital interventions but also the lived experiences of users, along with the facilitators of and barriers to long-term engagement. Previous studies have predominantly focused on short-term adherence and clinical outcomes [11], leaving limited insight into how patients with cardiac disease interact with self-monitoring platforms over extended periods, how usability and system design influence engagement, and whether continuous tracking supports or hinders long-term lifestyle behavior change. These gaps highlight the need for a more comprehensive understanding of how digital self-monitoring can be optimized for long-term adherence. Digital interventions have mainly focused on physical activity and diet, whereas interventions on other lifestyle factors such as sleep and stress management are less well studied [11] despite their well-documented impact on cardiovascular outcomes [16].

Therefore, we developed a lifestyle monitoring platform designed to balance accuracy, usability, engagement, and comprehensive tracking of key health behaviors over time. To achieve these goals, we carefully balanced the monitoring protocol to minimize patient burden while ensuring accurate insights into lifestyle behaviors based on the self-regulation theory [13], which emphasizes that individuals can manage behaviors effectively through goal setting, self-monitoring, and feedback. The platform was iteratively designed and refined through testing with both end users and experts in the field to enhance usability and engagement. We evaluated dropout and adherence patterns in patients with cardiac disease who were referred for or had undergone a cardiac intervention [17], and the results showed that over half of the patients used the system sustainably for 1 year following the intervention. These findings indicate that long-term lifestyle self-monitoring is feasible but that efforts should be made to increase patient engagement.

### Objectives

In this study, a mixed methods approach was used to identify factors that are related to long-term adherence results by evaluating the experiences of both completers and dropouts using both interviews and questionnaires. Specifically, this study investigated how patients experienced the usability of the system and how this differed between completers and dropouts. Second, key facilitators of and barriers to sustained self-monitoring were explored via interviews with completers of the study and reasons for discontinuation from dropouts. In addition, this study explored how completers perceived the impact of continuous self-monitoring on lifestyle awareness and long-term behavior change while also examining their overall experiences after the cardiac intervention to gain deeper insights into their recovery process and its potential impact on engagement. We also evaluated the system design choices to derive key learnings that can inform the development of future solutions. By integrating mixed methods data from both dropouts and completers, this study provides novel insights to optimize future digital health interventions for sustained engagement in long-term monitoring of lifestyle factors.

## Methods

### Study Design and Setting

This mixed methods study was part of a larger research project, the Cardiovascular Research Opting for New Applications (CARE-ON) project, which aimed to evaluate a custom-built integrated eHealth system combining a wearable sensor, a chatbot, and a dashboard for self-monitoring lifestyle behaviors in patients with cardiac disease. The project was carried out at the Máxima Medical Center (Eindhoven and Veldhoven, the Netherlands) in collaboration with the Eindhoven University of Technology, Department of Industrial Design. As part of the CARE-ON project, a 1-year prospective observational clinical trial was conducted from November 2021 to July 2024 to evaluate dropout, adherence, usability, and effects of the system on lifestyle behavior for patients referred for or who had recently undergone a cardiac intervention (a detailed description of the protocol is available elsewhere [17]).

### Study Equipment

**Figure 1:**
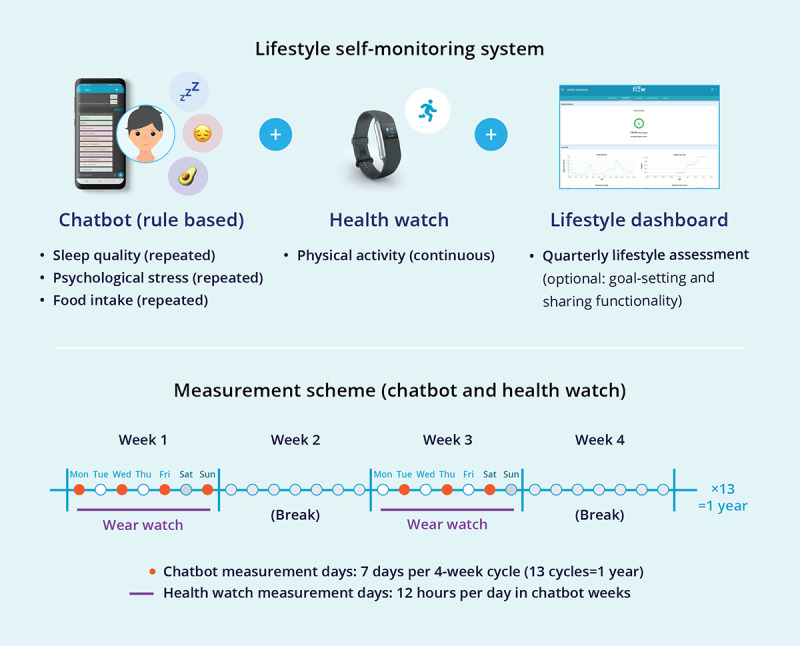
Illustration of the eHealth platform components and the measurement scheme for continuous lifestyle monitoring via chatbot prompts and health watch use.

The eHealth self-monitoring system consisted of 3 modules (Figure 1). The first module was a rule-based chatbot, which was accessible on both mobile and desktop, via which the patients received prompts to provide lifestyle data via a set measurement scheme (Figure 1). The chatbot was developed to capture habitual longitudinal self-report lifestyle data using mobile ecological momentary assessments with predefined questions and answer options. The chatbot focused on three domains, with questions based on validated tools: (1) sleep quality, based on the Consensus Sleep Diary [18] (only in the morning), (2) daily mental stress, based on the Positive and Negative Affect Schedule [19] (3 times per day), and (3) nutritional intake, using a 24-hour dietary recall method divided into 5 recall periods per day and integrated into the chatbot. This module was supported by a dietary database based on the Dutch Food Composition Database [20]. The development of the nutrition module is described in detail elsewhere [21].

**Figure 2:**
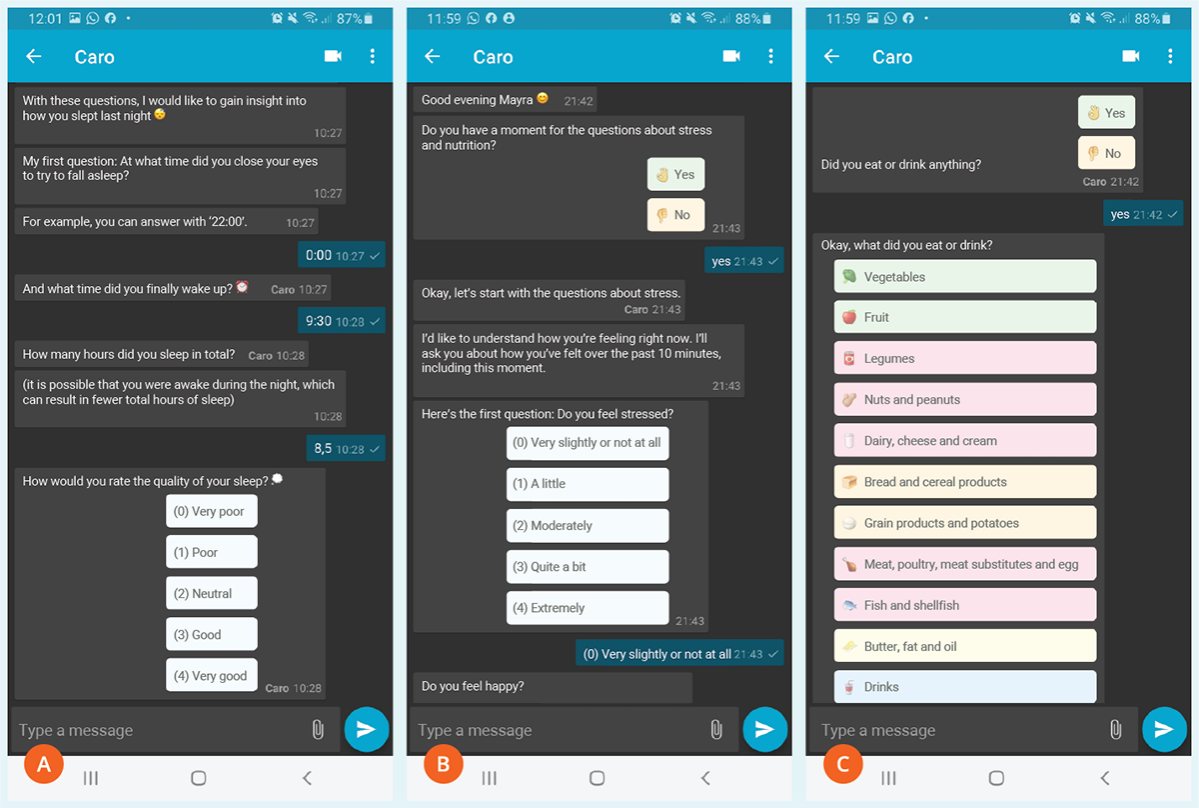
Screenshots of the rule-based chatbot—(A) questions related to sleep quantity and quality, (B) questions on psychological stress using a Likert scale, and (C) food categories available for users to select from when reporting food intake.

The patients received prompts in 4-week cycles: 7 measurement days over 2 weeks distributed across 3 to 4 days per week (2 to 3 weekdays and 1 weekend day) within a 4-week cycle. On designated measurement days, patients received 5 prompts per day, with the timing of these prompts personalized according to their individual daily routines. A more detailed overview of the chatbot modules, including sample question flows, is provided in Multimedia Appendix 1. Screenshots are provided in Figure 2.

The second module was an integrated health watch (Figure 1), with which physiological parameters (ie, steps, activity counts and type, heart rate, respiration rate, energy expenditure, sleep time, cardiovascular fitness index, and watch wear time) were continuously measured. Patients were asked to wear the health watch in the same weeks in which they received chatbot prompts.

**Figure 3:**
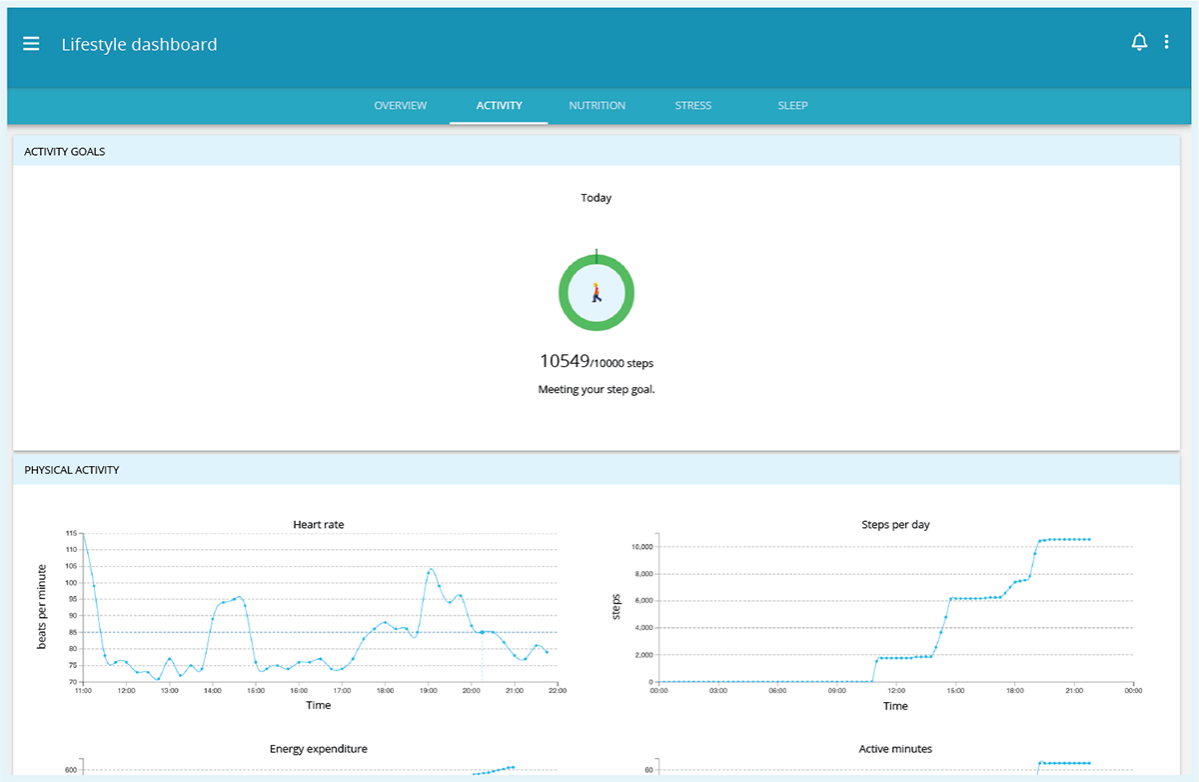
Screenshot of the desktop eHealth platform integrated with a health watch—lifestyle dashboard available to patients displaying health watch data and a standardized goal as visualized lifestyle feedback.

The third module was a platform where the collected data were visualized (Figure 3). In addition, lifestyle self-assessment questionnaires were made available quarterly via the platform, offering a point-in-time evaluation (that additionally included smoking behavior) and lifestyle advice based on the questionnaire scores. Patients could also use the platform to optionally set and monitor goals and share data with relatives or caregivers.

The patients were asked to at least use the chatbot and the health watch and fill in the quarterly lifestyle self-assessment questionnaires during 1 year for self-monitoring of their lifestyle behaviors.

### Ethical Considerations

The trial protocol was reviewed and approved by the institutional review board of the Máxima Medical Center, and the clinical trial was registered in the Dutch Trial Register (Landelijk Trial Register; registration NL9861). The CARE-ON clinical trial was conducted according to the Declaration of Helsinki. All patients provided written informed consent before study entry, including for the collection and analysis of the data used in this mixed methods study. Participant data were pseudonymized and stored securely to ensure confidentiality. No financial compensation was provided, although participants received a health watch on loan during the study period and access to the self-monitoring platform. The study protocol and the main results of the quantitative evaluation, including dropout and engagement over time, adherence, and usability, are described elsewhere [17,22]. The results are reported according to the Reporting of Studies Conducted Using Observational Routinely Collected Health Data [23] guidelines, which extend the Strengthening the Reporting of Observational Studies in Epidemiology statement, with the corresponding checklist provided in Multimedia Appendix 2.

### Recruitment and Study Procedures

The complete trial comprised a sample of 100 patients who were referred for or had recently undergone CABG; a (fractional flow reserve–guided) PCI; radiofrequency catheter ablation; or valve surgery, including transcatheter aortic valve implantation. Patients were recruited either when they were scheduled for the cardiac intervention or during their clinical admission following the procedure at the Máxima Medical Center. In cases in which only a diagnostic procedure (ie, fractional flow reserve test) was performed and no intervention was deemed necessary, patients were still included because of the initial treatment intent and their comparable clinical context. Patients were asked to participate by the study nurse either via a phone consultation or during a hospital visit after being referred for a clinical intervention by their treating physician.

After providing informed consent, patients received instructions on how to use the lifestyle self-monitoring platform as part of an intake procedure conducted by the researcher and nurse specialist. In addition to the baseline intake procedure, the research nurse or researcher contacted patients via quarterly phone interviews every 3 months (at 3, 6, 9, and 12 months) to inquire about medical events and gather feedback on the self-monitoring system. At the end of the study year, an evaluation interview was conducted with patients who completed the full year (completers). Patients who discontinued participation (dropouts) were contacted to provide a reason for dropout. At each of these time points (baseline, 3, 6, 9, and 12 months or at the time of dropout), patients were asked to complete quarterly lifestyle and other research-related questionnaires via the eHealth platform.

The interview questions of both the quarterly phone interviews and the evaluation interview were entered into the data collection software in advance. During the interviews, the researcher systematically asked the questions in the same order for all patients and directly transcribed their responses into the software. The phone interviews lasted between 5 and 10 minutes. The evaluation interview (the interview guide can be found in Multimedia Appendix 3) was conducted in person, and patients were interviewed individually (sometimes with relatives) at the hospital. Each interview lasted between 30 and 90 minutes. No audio recordings or verbatim transcripts were made as responses were documented in real time based on predefined questions, ensuring consistency across interviews and allowing for systematic data collection and analysis.

Health care providers did not have access to the collected data, and patients did not receive coaching based on their lifestyle data from the study nurse or researcher. Patients did receive structured chatbot notifications and reminders when a quarterly questionnaire was due.

Table 1 provides an overview of the data collection time points in addition to the lifestyle data captured via the self-monitoring system.

**Table 1:** Study setup and data collection time points.

Measurement		Study time point				
		Baseline—T0	Study year			
			3 mo	6 mo	9 mo	1 y (end)
**All study participants**						
	Quarterly phone interviews (measurement A) with study nurse		✓	✓	✓	
	Quarterly questionnaire (measurement B)—digital questionnaire via the platform		✓	✓	✓	✓
	End-of-study questionnaire (measurement C)—digital questionnaire via the platform (qualitative and quantitative)		✓^a^	✓^a^	✓^a^	✓
**Dropouts only**						
	Reasons for dropout (measurement D)—direct inquiry by study staff (nonmandatory)		✓^a^	✓^a^	✓^a^	✓^a^
**Study completers only**						
	Evaluation interview (measurement E)—structured interview with study staff (qualitative)					✓

^a^At the point of study dropout.

### Outcome Measures

Table 2 provides a more detailed overview of the outcome measures and their rationales, including the standard questions, questionnaires, and themes (particularly for the evaluation interview) used in this study.

**Table 2:** Outcome measures and rationales used in this mixed methods study.

Data source (qualitative or quantitative)	Rationale	Standard questions, questionnaires, and themes
Quarterly phone interviews (qualitative; measurement A)	Aimed at capturing usability challenges, system strengths and weaknesses, and potential enhancements, ensuring that patient experiences could be used to inform platform refinements	During these interviews, patients were asked to (1) report their experiences with the system in general, (2) report whether they experienced (technical) difficulties while using the platform, and (3) identify both strengths and weaknesses of the system.
Quarterly questionnaire (qualitative; measurement B)	Aimed at capturing usability challenges, system strengths and weaknesses, and potential enhancements, ensuring that patient experiences could be used to inform platform refinements	Open-ended question via the platform: “From your perspective, how can we improve the platform?”
End-of-study questionnaire (quantitative; measurement C)	To assess key TAM^a^ [24] components, including perceived usefulness (ie, the extent to which the system was seen as beneficial for health behavior change), ease of use (ie, the perceived effort required to interact with different platform components, such as the chatbot, dashboard, and health watch), and satisfaction with platform use	Perceived usefulness—one statement: “The feedback I receive from the patient platform helps me improve my health behavior.” Responses were collected using a 5-point Likert scale (1=“strongly disagree,” 2=“disagree,” 3=“neutral,” 4=“agree,” and 5=“strongly agree”). SEQ^b^—three SEQs [25] were used in this study: (1) answering chatbot questions, (2) collecting movement data using the health watch, and (3) viewing lifestyle data in the lifestyle dashboard. Responses were collected using a 7-point scale (where 1 indicated “very difficult” and 7 indicated “very easy”), with an additional option for nonuse. CSAT^c^ [26]—two questions based on the CSAT: (1) “How satisfied are you with this platform for the monitoring of lifestyle behavior?” and (2) “How satisfied are you with the ease of use and appeal of the platform?” Responses were collected with a scoring of 1 for “extremely dissatisfied,” 2 for “somewhat dissatisfied,” 3 for “neither satisfied nor dissatisfied,” 4 for “somewhat satisfied,” and 5 for “extremely satisfied.”
Dropout reasons (quantitative and qualitative; measurement D)	To gain deeper insights into the factors influencing disengagement	Direct inquiry by study staff. Providing this information was not mandatory.
Evaluation interview (qualitative; measurement E)	A structured evaluation interview explored patients’ experiences with posttreatment recovery, lifestyle changes, and self-monitoring behaviors (eg, facilitators of and barriers to long-term use), as well as their engagement with the system, including its usability (also per module) and perceived burden. This approach enabled a comprehensive assessment of the system’s feasibility, acceptability, and potential impact on long-term behavior change.	Post–cardiac intervention experiences: to contextualize engagement with lifestyle monitoring, we examined how patients’ recovery experiences influenced their use of the system. Effect of lifestyle self-monitoring on lifestyle awareness and behavior change: patients were asked to report on whether self-monitoring using the system had an effect on their lifestyle awareness and whether it helped them reflect on and eventually adjust their lifestyle behaviors in accordance with the self-regulation theory [13]. System usability and engagement: we investigated the overall usability of the system and how patients engaged with it. We evaluated key platform components, including chatbot interactions, wearable usability, feedback mechanisms, and goal-setting and sharing features. In addition, perceived effort and time investment regarding the proposed monitoring protocol were assessed using the NASA-TLX^d^ [27] scale.

^a^TAM: technology acceptance model.

^b^SEQ: single ease question.

^c^CSAT: customer satisfaction score.

^d^NASA-TLX: NASA Task Load Index.

### Data Analysis

Data analysis was structured around the study objectives, integrating quantitative statistical approaches and qualitative analyses. We describe the results based on 5 emerging themes.

The first theme is *system adoption and continued engagement*. This theme uses data from the end-of-study questionnaires (measurement C)—descriptive statistics were used to summarize central tendencies and variability. Independent-sample *t* tests (2-tailed), combined with the Cohen *d* to measure effect sizes, were used to assess group differences.

The second theme is *reasons for dropout: insights from patients who discontinued using the monitoring system*. Descriptive statistics were used to examine dropout reasons (measurement D), with frequencies and percentages calculated for each emerging theme to summarize reported reasons for discontinuation.

The third theme is *facilitators of and barriers to long-term engagement in self-monitoring of health behaviors*. Qualitative analyses involved an inductive coding approach (thematic analysis [28]) to identify key facilitators of and barriers to long-term self-monitoring of lifestyle behaviors drawing from all qualitative data sources (measurements A, B, D, and E). The final themes were structured into a coherent narrative to provide insights into facilitators of and barriers to long-term engagement.

The fourth theme is *effect of long-term lifestyle self-monitoring on lifestyle awareness and behavior change*. This theme aimed to examine the effects of long-term self-monitoring on lifestyle awareness and behavior change among completers. A structured analysis was conducted using interview data (measurement E) aligned with the self-regulation theory.

The fifth theme is *evaluation of usability of the lifestyle monitoring system design*. A structured approach was used to analyze patient-reported feedback on system usability, incorporating insights from all qualitative data sources (measurements A, B, D, and E). Responses were categorized based on predefined usability aspects, including system design and component usability (chatbot; health watch; dashboard, which primarily displays patients’ lifestyle data, but also includes the quarterly questionnaires, a goal-setting module [optional], and a sharing functionality [optional]). The NASA Task Load Index scores (measurement E), assessing the perceived burden related to self-monitoring, were analyzed quantitatively to assess the perceived burden of the monitoring protocol across interview participants.

Where relevant, qualitative findings were integrated with study outcomes to explore potential links among reported experiences, dropout, and adherence data from the system. In addition, when applicable, a deductive coding approach was used to structure qualitative findings within predefined themes.

## Results

### Study Sample

Of the 100 patients included in this study (mean age 61.6, SD 10.4 y; n=88, 88% male; n=45, 45% having undergone PCI; n=55, 55% having undergone another intervention), 57 (57%) completed the full study year, and 43 (43%) dropped out of the study. The demographic and clinical characteristics of the total sample, completers, and dropouts are described elsewhere [22]. In summary, no substantial differences were found between the groups with regard to sociodemographic characteristics, clinical history, and lifestyle factors at baseline.

Throughout the study, data were collected using five different methods: (1) quarterly phone interviews (measurement A); a total of 217 phone interviews were completed (n=46, 21.2% with dropouts and n=171, 78.8% with completers); (2) quarterly questionnaires (measurement B); a total of 157 quarterly questionnaires were administered (n=12, 7.6% to dropouts and n=145, 92.4% to completers); (3) end-of-study questionnaires (measurement C); a total of 77 patients (n=54, 70% completers; n=23, 30% dropouts) completed the end-of-study questionnaire; (4) reasons for dropout; of the 43 patients who dropped out, one died during the study; of the remaining 42 patients, n=41 (98%) provided a reason for their withdrawal; and (5) evaluation interviews: a total of 98% (56/57) of the completers took part in the evaluation interview. One completer was unable to participate in the evaluation interview for medical reasons.

### System Adoption and Continued Engagement (Theme 1)

**Figure 4:**
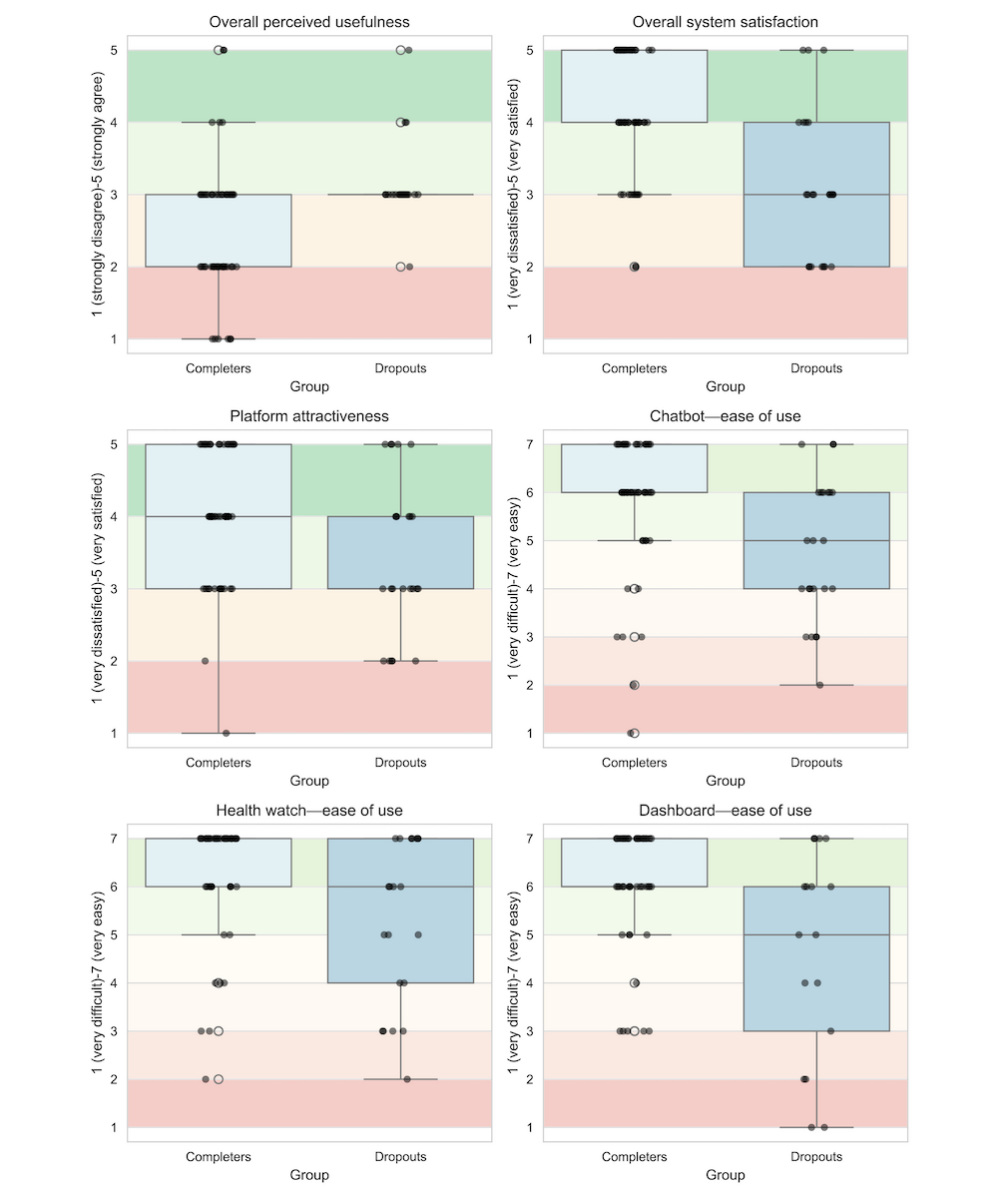
Box plots comparing perceived usefulness, overall satisfaction, platform attractiveness, and ease of use (chatbot, health watch, and dashboard), between completers and dropouts.

To examine differences in factors contributing to system adoption and sustained engagement in relation to the technology acceptance model, we analyzed end-of-study questionnaire responses from both completers and dropouts focusing on perceived usefulness and ease of use as key determinants of engagement. Figure 4 presents box plots comparing perceived usefulness, ease of use, and engagement-related usability ratings between the 2 groups. Background shading reflects interpretative ranges for each scale—satisfaction and agreement scores (1-5) follow a customer satisfaction score framework, where higher values (green) indicate positive experiences, midrange scores (gray) represent neutral responses, and lower values (red) indicate dissatisfaction. Ease of use scores (1-7) are visualized based on the single ease question scale, where green represents high ease of use, yellow indicates moderate difficulty, and red reflects increasing difficulty levels.

With respect to perceived usefulness, completers were significantly more likely to perceive the platform as beneficial, reporting that the feedback helped them improve their health behaviors (*P*<.001; *d*=0.75).

Regarding ease of use (chatbot, health watch, and dashboard), completers rated system usability significantly higher than dropouts, with chatbot ease of use showing the largest difference between groups (*P*=.02; *d*=0.61). Similar trends were observed for the health watch and dashboard, indicating that ease of interaction played a role in sustained engagement.

Finally, regarding platform satisfaction, completers reported significantly higher satisfaction with the platform than dropouts (*P*<.001; *d*=1.09). In addition, perceived platform attractiveness was lower among dropouts (*P*=.01; *d*=0.63), suggesting that visual and design aspects might have influenced engagement.

### Reasons for Dropout: Insights From Patients Who Discontinued Use of the Monitoring System (Theme 2)

#### Overview

In total, 43% (43/100) of the patients dropped out of the clinical trial and discontinued use of the self-monitoring system. On the basis of the quantitative analyses, we previously reported that the main reasons for dropout were high participation burden (22/43, 51%), including technical issues with the health watch and dissatisfaction with the chatbot, and personal reasons (19/43, 44%) [22]. In addition, one patient withdrew for undisclosed reasons, and another dropped out due to death. In this analyses, we identified the main reasons that were explicitly linked to the use of the self-monitoring platform (34/43, 79%) to clarify the underlying causes of discontinuation of use of the monitoring system.

The most frequently cited reason directly related to the self-monitoring platform was *burden of repeated self-reporting and dissatisfaction with the chatbot*, reported by 29% (10/34) of the patients. This was followed by *general usability issues across all modalities*, reported by 15% (5/34) of the patients. *Technical issues that led to frustration and demotivation* were cited by 9% (3/34) of the patients, and another 9% (3/34) identified *limited perceived usefulness* of the system as their primary reason for dropout. Among the personal reasons, 18% (6/34) of the patients reported that *self-monitoring caused mental stress*, whereas 15% (5/34) of the patients cited *low motivation*. The 6 key themes relevant to discontinuation of long-term monitoring of health behaviors are described in detail in the following sections.

#### Burden of Self-Reporting and Dissatisfaction

The chatbot was experienced as inefficient or requiring too much effort by 29% (10/34) of the patients, contributing to dissatisfaction and dropout. Patients found answering the same questions multiple times a day tedious, with some describing it as a routine that became a chore. A patient noted the following:

I’m also tired of having to answer the same questions three times a day, overkill.

Difficulties in correcting input errors, excessive notifications, and the inability to reuse previous responses (eg, for repeated meals) added to their frustration. Some found the system too time-consuming for proper completion, whereas others struggled with its lack of flexibility, such as the inability to accurately log a specific diet. Over time, certain questions became irritating, further diminishing motivation to continue.

#### Usability Issues Across All Modalities

A subset of patients (5/34, 15%) described perceived poor usability of the self-monitoring platform across a multitude of components of the monitoring system. These usability concerns limited their engagement and ultimately contributed to dropout. One patient expressed it as follows:

If everything had worked properly, I would have worn the watch longer and used the chatbot more often.

#### Technical Issues With the Health Watch Causing Frustration and Demotivation

Technical problems primarily involved synchronization issues and dissatisfaction with the health watch due to ergonomic concerns or malfunctions. A total of 9% (3/34) of the patients cited recurring synchronization failures as a key reason for dropout. These issues, unrelated to user error, stemmed from external system reliability problems, causing frustration and disengagement. One patient expressed their frustration stating the following:

I have not been able to sync for a week. These are all practical issues. My charger is acting up again. These things do not make it easy to keep up.

#### Perceived Lack of Usefulness

A subset of patients (3/34, 9%) questioned the added value of the self-monitoring system for their personal health management. When perceived benefits were unclear or not directly experienced, motivation to continue declined, leading some to disengage from the study. Patients stated the following:

I was already aware of my lifestyle, so I did not need this.

I realized I do not actually want to change my lifestyle.

#### Self-Monitoring Causing Mental Stress

In total, 18% (6/34) of the patients reported feeling overwhelmed by the repeated reminders and notifications, which created pressure to engage and added to their daily stress levels. Some (2/34, 6%) became overly self-critical or anxious due to continuous self-monitoring. A patient noted the following:

It became an obsession; I kept checking my heart rate and panicked whenever I saw an unusual reading.

I’m too focused on the medical aspects of the watch, and that does not benefit my health.

Others (4/34, 12%) felt that the frequent prompts made their illness too present in daily life, making it harder to focus on living normally. One patient put it as follows:

There are too many reminders of my illness when I just want to live.

#### Low Motivation

Low motivation was cited by 15% (5/34) of the patients, with some stating that they were not engaged from the start or frequently forgot about the study components. One patient mentioned the following:

I find it hard to motivate myself to do this. I forget the watch, the questionnaires, and I’m not good at keeping up with the chatbot.

These responses suggest that personal motivation and perceived benefit played a role in dropout.

### Facilitators of and Barriers to Long-Term Engagement in Self-Monitoring of Health Behaviors (Theme 3)

Understanding the factors that influence long-term engagement with digital self-monitoring is critical for optimizing usability and adherence. We analyzed open-text responses from quarterly phone interviews, questionnaires (quarterly and at the end of the study), reasons for dropout, and the evaluation interviews to identify key facilitators and barriers. Table 3 presents the overarching themes: usability and technical aspects, motivation and perceived usefulness, psychological factors, lifestyle behavior change, external and social influences, and customization and adaptability. The facilitators highlight features that encouraged continued participation, such as minimal-effort tracking, perceived health benefits, and habit formation. Conversely, the barriers reflect obstacles that led to discontinuation of use of the monitoring system, including technical frustrations, psychological burden, and lack of perceived relevance.

**Table 3:** Factors influencing adherence to the self-monitoring system.

Theme	Facilitators	Barriers
Usability and technical aspects	Minimal effort required for wearables (passive data collection) Perceived ease of use of some components (dashboard and step tracking) System reliability when functioning properly Integrated within the care platform, ensuring security without the need for multiple apps	Technical frustrations (synchronization failures and inaccurate readings) Repetitive chatbot questions Issues with usability, reminder preferences, and navigation Battery life concerns with wearables Discomfort (eg, skin irritation) Already owning a better functioning device that could not be integrated Limited abilities of the watch
Motivation and perceived usefulness	Perceived benefit of self-monitoring for health insights Positive reinforcement from tracking progress Goal setting motivation (for some patients)	Low perceived personal relevance No immediate feedback or tailored insights Lack of motivation to engage with the system
Psychological factors	Habit formation over time (routine integration) Sense of accountability and self-awareness	Psychological burden from continuous tracking (stress and anxiety) Self-monitoring fatigue (overwhelming number of reminders) Frustration with tracking unexpected fluctuations (eg, heart rate spikes)
Lifestyle behavior change	Increased awareness of physical activity and nutrition Some behavior modifications based on self-monitoring	Minimal impact of self-monitoring on stress awareness and management Limited engagement with lifestyle goal-setting features
External and social influences	Support from the study team and research nurse Social support from partner and social environment	Competing priorities (work and personal life distractions) Interruptions due to holidays, travel, or daily schedules Preference for wearing a different-looking watch for special occasions
Customization and adaptability	Flexibility in wearable use (passive tracking) Structured chatbot reminders (eg, notifications) The ability to configure and adjust the timing of chatbot prompt notifications to align with individual routines	Limited chatbot personalization (eg, saving meals)

Habit formation and reminders supported chatbot adherence, whereas boredom, frustration, and forgetfulness were associated with discontinuation. The health watch provided a sense of security, but technical issues and inaccurate measurements sometimes led to frustration. Data reliability issues such as heart rate outliers, not registering certain activities, and missing step counts affected patient trust in the system. The practicality of adherence was influenced by technical challenges (eg, synchronization issues and notification failures) and usability concerns (eg, log-in barriers, skin irritation, and already owning another smartwatch). However, adherence was also supported by a sense of control over heart rate monitoring, the feeling of being observed and accountable, and the availability of study team support when needed.

### Effect of Long-Term Lifestyle Self-Monitoring on Lifestyle Awareness and Behavior Change (Theme 4)

#### Overview

This section examines the experiences of study completers who engaged in self-monitoring of lifestyle behaviors using the monitoring system throughout the year following their cardiac intervention. Their postintervention recovery experiences were explored first. Understanding this process is crucial as it can influence their engagement with lifestyle changes and the sustainability of healthy behaviors over time. Furthermore, based on the evaluation interviews, the findings provide insights into how self-monitoring influenced lifestyle awareness and sustained behavior change framed within the context of the self-regulation theory.

#### Postintervention Recovery: Physical and Mental Adaptation in Daily Life

To better understand patients’ engagement with the self-monitoring system, their postintervention recovery experiences were explored. While most reported full physical recovery, mental adjustment varied. To capture these differences, recovery experiences were categorized into physical and mental dimensions, as shown in Table 4. For physical recovery, 3 categories were defined: fully recovered (patients whose physical health and functionality returned to preintervention levels or who felt fully returned to normal), improving or partially recovered (those who made noticeable progress but were not yet fully recovered), and no improvement or deterioration (patients with no improvement or a decline in physical condition). A parallel framework was applied to mental recovery, with categories labeled as accepting (patients who came to terms with their new reality and showed emotional stability), improving (those gradually adapting and showing mental health improvement), and struggling (those facing ongoing emotional or psychological challenges after the cardiac intervention). These categories provided insights into how patients managed their recovery and how it related to their engagement in self-monitoring.

Patients who reported full physical recovery (38/56, 68%) exhibited the highest engagement with both the chatbot and health watch, with only 31% (12/38) showing low chatbot adherence and 11% (4/38) showing low health watch adherence. In contrast, those who experienced no improvement or deterioration (6/56, 11%) were more likely to disengage, with 67% (4/6) showing low chatbot adherence and, similarly, 67% (4/6) showing low health watch adherence. A similar trend was observed in mental recovery—patients who fully accepted their condition (37/56, 66%) demonstrated more balanced adherence patterns, whereas those struggling (5/56, 9%) had the highest rates of low chatbot adherence (3/5, 60%), but lower rates of low health watch adherence (1/5, 20%). Notably, chatbot adherence appeared more affected by recovery status than health watch adherence, suggesting that passive data collection was easier to maintain than active chatbot interaction.

Patients’ diagnosis and the type of cardiac intervention were associated with recovery experiences. Patients recovering from myocardial infarction, particularly those treated with PCI, generally reported better recovery and higher adherence. In contrast, those experiencing persistent dyspnea, often following valve surgery, showed lower adherence. While most patients (38/56, 68%) reported full physical recovery, many still noted reduced fitness, attributing it to aging and overall health decline. Slower recovery was more common among patients who had undergone valve surgery, whereas a small group (6/56, 11%) saw no improvement or deterioration, often adjusting their daily activities by slowing their pace and taking more breaks.

Mental recovery showed a more complex pattern of results (Table 4). Increased awareness of their condition led some patients to experience anxiety, heightened sensitivity to bodily sensations, and emotional strain. While most ultimately accepted their situation and adapted, others remained cautious, avoiding activities due to fear of overexertion. A small group (5/56, 9%) struggled emotionally, exhibiting reduced activity, forgetfulness, and changes in self-perception. Younger patients appeared to have more difficulty adjusting mentally than older patients, suggesting that age might play a role in psychological adaptation. Many described experiencing a “turning point” in their recovery, after which they moved toward acceptance, regaining confidence and emotional stability. While physical recovery was often seen as a more straightforward process, mental adaptation required more time and emotional adjustment.

**Table 4:** Overview of post–cardiac intervention experiences of the interviewed patients (completers).

Postintervention experience			Diagnosis		Intervention or procedure		Adherence level		Example quotes
**Physical recovery**									
	Fully recovered: 38 (68%); mean age 62.9 (SD 9.7) y; 35 (92%) male	Most common: MI^a^ (n=15, 40%); other diagnoses: AF^b^, AP^c^, atrial flutter, dyspnea, arrhythmia, and no symptoms		Most common: PCI^d^ (n=17, 45%); other interventions or procedures: CABG^e^, FFR^f^, RFCA^g^, valve surgery, and other		Chatbot: 12 (31%) low, 13 (34%) moderate, and 13 (34%) high; health watch: 4 (11%) low, 6 (42%) moderate, and 28 (74%) high		“I do not see myself as a heart patient. It was a technical malfunction [related to the heart] and that has been resolved.”	
	Improving or partially recovered: 12 (21%); mean age 61.4 (SD 11.9) y; 9 (75%) male	Most common: dyspnea (n=7, 58%); other diagnoses: AF and AP		Most common: valve surgery (n=7, 58%); other interventions or procedures: CABG, PCI, and RFCA		Chatbot: 4 (33%) low, 4 (33%) moderate, and 4 (33%) high; health watch: 1 (8%) low, 3 (25%) moderate, and 8 (67%) high		“It feels like the brakes are on [walking with resistance] when I go for a walk.”	
	No improvement or deterioration: 6 (11%); mean age 63.7 (SD 8.2) y; 5 (83%) male	Most common: AP (n=2, 33%) and MI (n=2, 33%); other diagnoses: dyspnea and no symptoms		Most common: PCI (n=4, 67%); other interventions or procedures: CABG and valve surgery		Chatbot: 4 (67%) low, 1 (17%) moderate, and 1 (17%) high; health watch: 1 (17%) low, 1 (17%) moderate, and 4 (67%) high		“Before the treatment, I felt better than I do now. My physical fitness has deteriorated significantly, and that is unpleasant.”	
**Mental recovery**									
	Accepting: 37 (66%); mean age 63.3 (SD 9.3) y; 33 (89%) male	Most common: MI (n=10, 27%); other diagnoses: AF, AP, atrial flutter, dyspnea, arrhythmia, and no symptoms		Most common: PCI (n=16, 43%); other interventions or procedures: CABG, FFR, RFCA, and valve surgery		Chatbot: 13 (35%) low, 12 (32%) moderate, and 12 (32%) high; health watch: 4 (11%) low, 6 (16%) moderate, and 27 (73%) high		“I’m doing very well. I came here by bike. You can feel tired, but you just push through. Just keep your head up and keep going. I do not want to be part of the older generation yet.”	
	Improving or adjusting: 14 (25%); mean age 61.9 (SD 12.8) y; 12 (86%) male	Most common: MI (n=5, 36%); other diagnoses: AF, AP, dyspnea, and no symptoms		Most common: PCI (n=6, 43%); other interventions or procedures: CABG, RFCA, valve surgery, and other		Chatbot: 4 (29%) low, 5 (36%) moderate, and 5 (36%) high; health watch: 1 (7%) low, 4 (29%) moderate, and 9 (64%) high		“My daily life has not changed much, but mentally it has.” “I’m not playing team sports yet. There is a voice in my head saying, ‘be careful.’”	
	Struggling: 5 (9%); mean age 60.0 (SD 4.74) y; 4 (80%) male	Most common: dyspnea (n=2, 40%) and MI (n=2, 40%); other diagnosis: AP		Most common: PCI (n=2, 40%) and valve surgery (n=2, 40%); other interventions or procedures: CABG		Chatbot: 3 (60%) low, 1 (20%) moderate, and 1 (20%) high; health watch: 1 (20%) low, 0 (0%) moderate, and 4 (80%) high		“I do everything with less effort, because I fear that it might happen again.”	

^a^MI: myocardial infarction.

^b^AF: atrial fibrillation.

^c^AP: angina pectoris.

^d^PCI: percutaneous coronary intervention.

^e^CABG: coronary artery bypass graft.

^f^FFR: fractional flow reserve.

^g^RFCA: radiofrequency catheter ablation.

#### Lifestyle Awareness and Behavior Change Through Long-Term Self-Monitoring

In this section, we examine how patients engaged with their data, reflected on their progress, and translated insights into lasting lifestyle changes, aligning with self-regulation theory, which emphasizes the role of self-monitoring in enhancing awareness, setting goals, and adapting behavior. Table 5 summarizes patients’ experiences with self-monitoring of key lifestyle behaviors, including physical activity, nutrition, sleep, and stress management. For each lifestyle behavior, 2 areas of experiences are described: *awareness and reflection* and *behavior change and adaptation*.

The self-monitoring system primarily enhanced lifestyle awareness, particularly regarding physical activity, nutrition, and, to a lesser extent, sleep, making patients more conscious of their movement patterns, food choices and portion sizes, and sleep quality, respectively. However, sustained behavior change varied depending on individual motivation, preexisting habits, and the perceived need for change. While some patients increased their physical activity and adopted healthier eating habits, others used self-monitoring more for validation than for modification. Notably, stress self-monitoring had the least impact, with only 1 patient reporting increased awareness, and no evidence of behavior change in this area was found. The findings underscore the role of self-monitoring in self-regulation—while it can support reflection and goal-directed behavior, its effectiveness depends on individual engagement, personal circumstances, and the cognitive burden of active self-reporting.

**Table 5:** Impact of self-monitoring on lifestyle awareness and behavior change—patient-reported experiences and outcomes.

Lifestyle behavior and experiential dimension			Patients (n=56), n (%)		Impact of self-monitoring on lifestyle awareness		Example quotes
**Physical activity**							
	Awareness and reflection	25 (45)		Increased awareness of movement patterns Monitored steps and heart rate		“I was aware of my steps and movement, and that worked well for me.”	
	Behavior change and adaptation	16 (29)		Motivated to move more Increased intentional activity choices Sustained change in daily activity levels		“If I was at 3000 steps, I made sure to add another 3000.” “Walking felt less intense over time, which was a good thing.”	
**Nutritional intake**							
	Awareness and reflection	29 (52)		Conscious of food choices and portion sizes Awareness of nutritional balance		“Through the chatbot, I became more aware of what I eat and how much I eat.”	
	Behavior change and adaptation	16 (29)		Reduced unhealthy food intake More deliberate food selection Long-term changes in eating habits, such as switching to healthier alternatives		“I swapped bread for dairy, and I drink a little less wine now.” “I eat smaller portions because I have to log them; it stays in my mind.”	
**Sleep quality**							
	Awareness and reflection	7 (13)		Greater awareness of sleep patterns and rest quality		“Seeing my sleep graph helped me realize when my rest was disturbed.”	
	Behavior change and adaptation	4 (7)		Adjusted bedtime habits to improve sleep duration		“I started going to bed earlier and making sure I got enough sleep.”	
**Mental stress levels**							
	Awareness and reflection	1 (2)		Awareness of general mood states		“When I have to fill in the mood states, I do think about it.”	
	Behavior change and adaptation	0 (0)		—^a^		—	

^a^Not applicable.

### Evaluation of Usability of the Lifestyle Monitoring System Design (Theme 5)

#### Overview

Beyond usability and engagement, this study examined how specific design choices shaped user experiences. Table 6 summarizes each design choice, intended function, user feedback from completers and dropouts, and recommendations for improvement. These insights provide practical guidance for optimizing digital health interventions to enhance long-term adherence and usability.

User engagement varied, with accessibility, personalization, and technical reliability as key factors. On the basis of patient feedback, thorough onboarding to all system components is recommended to facilitate use and enhance overall user experience. The chatbot was motivational but felt repetitive and complex, requiring adaptive personalization. The health watch provided valuable insights but faced technical issues. Quarterly assessments and information on the dashboard were informative (“I checked if I was being ‘green’ [green refers to a healthy food choice, as indicated by a green traffic light score]”) but would benefit from a mobile version and, for the dashboard, more personalized real-time feedback. The goal-setting functionality had low engagement, requiring simpler guidance (“Setting a new goal was complicated”). The data sharing functionality was unused, needing clearer benefits and privacy assurances.

**Table 6:** Impact of design choices on usability and engagement.

Design feature (modality and engagement of completers)	Intended function	Positive user feedback	Negative user feedback	Effectiveness and recommendations
Chatbot interactions (mobile; mandatory component; engaged completers: n=57, 100%)	Support structured self-monitoring and lifestyle reflection	It was valued for its ease of use, usefulness, motivational impact, and ability to create awareness. In addition, habit formation, such as integrating it into daily routines (eg, notification timing aligned with personal schedule), was appreciated.	It was perceived as repetitive, complex (specifically with respect to nutrition), and difficult to correct, leading to reduced engagement over time. Users occasionally forgot to respond to prompts, and stress-related questions felt redundant.	Needs adaptive personalization to maintain engagement.
Health watch (device; mandatory component; engaged completers: n=57, 100%)	Passive lifestyle tracking (eg, heart rate, activity, and sleep)	It was valued for its heart rate insights, step tracking, motivational impact, sleek design, ease of use, and overall usefulness.	Frustration arose from technical issues, including synchronization failures, measurement inaccuracies, and short battery life.	Ensure technical reliability and offer alternative device options.
Quarterly lifestyle self-assessment via the dashboard (desktop web application; mandatory component; engaged completers: n=57, 100%)	Self-assessment tool for cardiovascular risk behaviors and providing feedback via the dashboard	It was valued for its immediate results and comprehensiveness, as well as the (sometimes strict) feedback and advice provided at the end of the questionnaires.	Limited accessibility due to desktop-only version. Questionnaires were lengthy but manageable.	Improve accessibility by offering mobile version.
Lifestyle dashboard (desktop web application; engaged completers: n=32, 58%)	Encourage behavior change through reflecting on self-monitoring insights	It was valued for useful nutritional advice (indication of healthiness per food item) and other health insights.	Limited accessibility (desktop only) and absence of real-time feedback reduced usability.	AI^a^-driven dynamic feedback could enhance engagement. Users expressed a desire to compare their health status with normative data for their patient group. Improving accessibility through a mobile app is recommended.
Goal-setting functionality (optional; desktop web application; engaged completers: n=4, 7%)	Allow users to set and track personal lifestyle goals	Gave motivation to set own goals or positively adjust goals (eg, increased steps).	Limited engagement due to lack of guidance and complexity.	Offer clearer instructions and integrate automated, personalized goal recommendations to improve usability.
Sharing functionality (optional; desktop web application; engaged completers: n=0, 0%)	Enable users to share collected lifestyle data via the platform with relatives or health care professionals	—^b^	No engagement due to lack of guidance, lack of perceived benefit, and privacy concerns.	Provide clearer use cases, privacy assurances, and user education on potential benefits.

^a^AI: artificial intelligence.

^b^Not applicable.

#### Perceived Effort of the Monitoring Protocol

The perceived effort of adhering to the proposed monitoring protocol was assessed through a NASA Task Load Index question on required effort among the interviewed patients (n=56). In total, 75% (42/56) of the patients indicated that only low effort was required to adhere to the protocol of the study, whereas 18% (10/56) of the patients indicated that it required medium effort, and 7% (4/56) indicated that it required high effort. Overall, the system’s protocol was perceived as relatively easy to integrate into patients’ daily lives because of its user-friendly interface, ease of integration into daily routines, immediate feedback, and accessibility on multiple devices. However, the perceived burden was significant for some patients, primarily related to the sustained effort required, the complexity and length of the questionnaires, occasional technical issues, and challenges in integrating certain components (such as the chatbot) into their routines. While the system was generally well received, these burdens impacted long-term engagement and satisfaction for some users.

## Discussion

### Principal Findings

#### Overview

This study provides insights into the use of a long-term digital lifestyle self-monitoring system in routine cardiac care. By evaluating patients’ experiences over 1 year after a cardiac intervention, we identified key factors influencing engagement and dropout. Completers reported significantly higher perceived usefulness, ease of use, and satisfaction than dropouts, highlighting the importance of a positive user experience. Dropouts commonly cited dissatisfaction with the chatbot, technical issues with the health watch, and the burden of self-reporting, whereas completers found the protocol to be low effort. Facilitators of adherence included routine formation, structured reminders, and the ease of using the wearable. Barriers included cognitive load from repetitive chatbot questions, persistent technical problems, and physical or emotional recovery challenges. Patients experiencing poorer recovery were more likely to disengage. While self-monitoring raised awareness, especially regarding physical activity and nutrition, it had limited influence on actual behavior change, suggesting a need for improved system features and support.

#### Engagement and Dropout: Balancing Usefulness, Burden, and Technical Trust

Engagement with digital self-monitoring tools depends on perceived usefulness, usability, emotional impact, and technical reliability. In this study, patients who completed the monitoring year were more likely to find the system valuable, motivating, and easy to use, findings that aligned with the technology acceptance model and findings of previous research [29,30] emphasizing that perceived usefulness and ease of use are key to sustained engagement. In contrast, dropouts often perceived the system as redundant or frustrating, particularly when they already considered their lifestyle to be healthy and saw limited added value in self-monitoring.

Burden and psychological strain also contributed to dropout. Excessive notifications, repetitive chatbot dialogues, and the perceived obligation to self-report created monitoring fatigue. For some patients, frequent reminders of illness heightened stress or anxiety rather than offering support, underscoring previous research indicating that self-monitoring can be empowering for some but distressing for others [31,32].

Technical frustrations, particularly with health watch reliability and chatbot inefficiencies, were another major barrier to long-term engagement. Unlike interface usability issues, which users can often adapt to, recurring connectivity failures eroded trust and motivation. Consistent with previous research [33,34], our findings suggest that confidence in digital health tools depends on the availability of accurate, real-time data. When data are lost or inconsistent, user trust declines. These issues highlight the importance of seamless technical integration, intuitive troubleshooting, and transparent communication about system limitations supported by user education on how to interpret and act on health data.

#### Personalization and Design Considerations for Sustained Engagement

To support long-term use, future systems should adopt adaptive strategies such as personalized chatbot interactions, flexible reminder schedules, and predictive analytics. These approaches can help reduce monitoring fatigue and emotional resistance while enhancing perceived relevance, value, and trust in the system. Personalization in particular emerged as a promising strategy to increase engagement and reduce dropout. Evidence shows that health chatbots tailored to user profiles through personalized content, interfaces, or communication styles improve user satisfaction and dialogue quality [35-37]. This may include aligning messages with a patient’s medical history, goals, or preferences; adapting interface elements such as avatars or notification frequency; and adjusting language to suit varying levels of health literacy or emotional tone. Granting users control over message timing or tone may further enhance comfort and reduce stress [38]. Personalized coaching interventions have shown positive effects on adherence across health domains [39]. Similarly, a chatbot could learn a user’s preferred communication style [40] or detect behavioral lapses such as reduced physical activity and respond with tailored encouragement or barrier exploration. In practice, this might involve an initial intake procedure to understand the patient’s context followed by ongoing adaptation based on user behavior over time. Personalization not only increases perceived relevance and satisfaction but may also enhance adherence and long-term engagement.

In addition to personalization, system design features played a critical role in shaping engagement in this study. Passive tracking was a strong facilitator, but recurring technical issues, particularly with the health watch, often undermined these benefits. While scheduled chatbot interactions helped some users establish healthy routines, others found the repetitive nature of dialogues demotivating. Furthermore, the lack of real-time feedback limited the platform’s ability to maintain user interest. Previous research [41] suggests that dynamic, personalized feedback supports adherence, reinforcing the need for artificial intelligence–driven personalization that adapts system behavior to individual needs and preferences. These findings highlight the importance of thoughtful, user-centered design that integrates reliability, adaptability, and relevance to support long-term digital health engagement.

#### Post–Cardiac Intervention Recovery and Engagement

Although this was not a primary focus, our study showed that the patients’ recovery trajectory influenced engagement. Most completers reported full physical recovery especially after PCI, whereas others faced ongoing symptoms or limited improvement. Interestingly, rapid recovery (eg, after PCI) can sometimes lead to complacency as patients may perceive the intervention as a definitive fix, reducing motivation for continued self-care or cardiac rehabilitation [42]. In contrast, those undergoing more invasive procedures such as CABG often face a more gradual and challenging recovery, which can increase engagement with structured rehabilitation programs to rebuild physical capacity [43]. Nevertheless, we found no significant differences in adherence by intervention type. In our study, recovery experiences rather than the procedure itself were more strongly associated with engagement. These differences suggest that perceived need for continued care influences engagement with digital tools.

Not only physical but also mental recovery varied across patients—while many recovered well, some struggled emotionally. Differences in psychological recovery have also been reported to influence engagement. Research consistently indicates that emotional factors such as stress, anxiety, and perceived burden negatively impact long-term adherence to lifestyle changes [39,44]. More specifically, patients experiencing postintervention anxiety may avoid self-monitoring to reduce stress, whereas those who reach a state of acceptance are more likely to perceive monitoring as beneficial rather than burdensome [45]. Thus, building on the earlier personalization theme, support strategies should also address individual physical and psychological recovery trajectories. Adaptive interventions such as flexible logging schedules and motivational feedback can support users through difficult periods and improve adherence. Tailoring the system to the patient’s recovery stage, emotional readiness, and capacity allows for more individually tailored, effective support in cardiac rehabilitation.

#### Self-Monitoring and Behavior Change: A Self-Regulation Perspective

Self-monitoring has been shown to be a tool for self-regulation [46], increasing patients’ awareness of their lifestyle habits, particularly regarding physical activity and nutrition. In our study, completers indeed reported more positive behavior change in these areas. However, the system had limited impact on stress management. Patients under psychological strain often saw tracking as burdensome, which aligns with our previous findings [22] showing that depressive symptoms and lower mental quality of life predict low adherence to self-monitoring interventions. This is also consistent with findings suggesting the necessity of incorporating psychological support and adaptive personalization to maintain engagement among populations vulnerable to emotional distress [32,47]. This reflects the need for integrated psychological support and mental health–sensitive design in digital interventions. Features such as goal setting, timely feedback, and mental health tailoring can foster more sustainable behavior change.

### Limitations

One key limitation is the absence of more thorough dropout perspectives as interviews were only conducted with completers. Including those who disengaged could have enriched the understanding of barriers such as low perceived benefit, poor usability, or external life factors. Future research should aim to include interviews with both completers and dropouts to gain a more comprehensive understanding of the factors influencing participation and sustained use.

Another limitation is the lack of audio recordings or verbatim transcripts as responses were documented in real time based on predefined questions by a single interviewer. The absence of verbatim records may have limited the depth of qualitative insights and may have hindered the ability to capture nuanced expressions or spontaneous remarks. While the approach facilitated efficient data processing and reduced the potential for subjective interpretation during transcription, it may have introduced interviewer bias. In addition, the structured interview approach may have constrained the emergence of unexpected insights, whereas recall bias could have influenced participants’ reflections on experiences that occurred weeks or months earlier. Moreover, participants may have been influenced by social desirability bias.

The study sample may not fully represent the broader population of patients with cardiac disease as participants were relatively homogeneous. As a result, the findings may have limited generalizability to other groups, such as those with lower digital literacy or differing health backgrounds.

### Conclusions

This study identified critical factors that influence long-term use of digital lifestyle self-monitoring systems in cardiac care. Completers found the platform more useful, more user-friendly, and less effortful than dropouts, who cited chatbot dissatisfaction and health watch technical issues. While self-monitoring increased awareness, particularly regarding physical activity and nutrition, its impact on behavior change was limited. To maintain engagement, systems must reduce burden; support routine formation; and offer personalized, psychologically attuned experiences. Ultimately, by improving usability, minimizing complexity and technical barriers, enhancing personalization, and providing psychological support, digital health tools can be optimized for long-term engagement, potentially promoting sustained positive health behavior change in cardiac rehabilitation and beyond.

## Appendix

Multimedia Appendix 1 Sample Flows of the Rule-Based Chatbot.

Multimedia Appendix 2 The Reporting of Studies Conducted Using Observational Routinely Collected Health Data (RECORD) statement (extension of the Strengthening the Reporting of Observational Studies in Epidemiology (STROBE) statement) Checklist.

Multimedia Appendix 3 Structured Interview Guide.

## Abbreviations

CABG: coronary artery bypass graft
CARE-ON: Cardiovascular Research Opting for New Applications
PCI: percutaneous coronary intervention

## Data Availability

The datasets generated or analyzed during this study are not publicly available due to inclusion of sensitive information, but they are available from the corresponding author upon reasonable request.
