# Supplementary material for: Patient-Reported Experiences With Long-Term Lifestyle Self-Monitoring in Heart Disease: Mixed Methods Study
Source: JMIR Form Res. 2025 Sep 17;9:e76978. doi: 10.2196/76978 (PMC12489404; doi:10.2196/76978)
Supplement: Multimedia Appendix 1 [file formative_v9i1e76978_app1.docx]

## Multimedia Appendix 1: Sample Flows of the Rule-Based Chatbot

An example schedule of the chatbot prompts on a measurement day, along with a detailed overview of the questions for each lifestyle parameter, including the recall period and number of items, is provided below.

| **Schedule** | **Example time^a^** | **Chatbot modules** |
| --- | --- | --- |
| At wake up | 08:00 | Sleep and Stress |
| After breakfast | 10:00 | Nutrition |
| After lunch | 13:00 | Nutrition and Stress |
| After diner | 20:00 | Nutrition and Stress |
| Morning after^b^ | 08:00 | Nutrition |
| ^a^Users could set the prompt timings according to their personal routines. They were advised to maintain at least a two-hour interval between the “wake-up” and “breakfast” prompts.  ^b^On the following morning, patients were asked to report only what they had consumed the previous day, from final prompt until bedtime. | | |

| **Sleep Quality** | |
| --- | --- |
| Recall period: | Once in the morning |
| Items: | 5 |
| 1. What time did you try to go to sleep? (answer type: time input) 2. What time was your final awakening? (answer type: time input) 3. In total, how long did you sleep? (answer type: numeric input—hours) 4. How would you rate the quality of your sleep?  (answer options: 1=“very poor,” 2=”poor,” 3=“fair,” 4=”good,” and 5=”very good”) 5. How restful or refreshed did you feel when you woke up for the day?  (answer options: 1=”not at all rested,” 2=”slightly rested,” 3=”somewhat rested,” 4=”well-rested,” and 5=”very well-rested”) | |

| **Mental Stress Levels** | |
| --- | --- |
| Recall period: | 3 times per day |
| Items: | 9 |
| We would like to know how you are feeling. Please indicate how you felt over the past 10 minutes, including now.  Response format: 5-point Likert scale (1=”not at all or very slightly,” 2=”a little,” 3=”moderately,” 4=”quite a bit,” and 5=”extremely”)   1. Stressed 2. Happy 3. Irritated 4. Energetic 5. Anxious 6. [Patient tailored feeling]^a^ 7. Do you feel in control of what you need to do? 8. Do you feel that a lot is being demanded of you? 9. I felt physically well    1. (if 2 or higher, the following questions are asked): what is bothering you? (answer type: open text)    2. How worried are you about this? | |
| ^a^The ‘patient tailored feeling’ is a custom item specific to each patient, which is asked at baseline. | |

| **Nutritional Intake** | |
| --- | --- |
| Recall period: | 4 times per day (2–3-hour recall period) |
| Items: | N/A |
| During the past [period of time], what did you eat or drink?  Answer type: buttons/numbers, or open text^a^.  Categories provided from the food composition database:     🥦 Vegetables    🍎 Fruit  🌽 Legumes  🥜 Nuts and peanuts  🥛 Dairy, cheese and cream  🍞🍝🍚 Bread, cereal products and potatoes  🍖 Meat, meat substitutes and egg  🐟 Fish and shellfish  🧈 Butter, fat and oil  ☕️🍵🥤🍺 Drinks  🧂 Salt  🍲 Soup  🍫 Sugar and confectionery  🍰 Cake and pastry  🍟 Savory snacks and fast food  These categories are followed by consecutively related questions:   1. Food item level: specify food item levels (eg, what kind of meat), based on predefined food items from the food database^b^.  (answer type: predefined buttons) 2. Portion size (corresponding to food item) and number of portions. (answer type: predefined buttons, numeric input for number of portions)    - In addition, related questions regarding the possible associated foods (eg, spreads, sugar, fat or salt) added to the reported food item are initiated accordingly. 3. After adding each food item, the chatbot reports back to the users in the chat and checks if they need to alter their input in case of misreporting. 4. When having reported an item, the chatbot loops back until the patient has reported all consumed food items for the specified recall period. | |
| ^a^Fuzzy terminology recognition was incorporated exclusively in this chatbot module to enable the use of natural language inputs.  ^b^If a food item was not available in the food database, users were instructed to select the item most similar to the one they had consumed. | |
