# Supplementary material for: Patient-Reported Experiences With Long-Term Lifestyle Self-Monitoring in Heart Disease: Mixed Methods Study
Source: JMIR Form Res. 2025 Sep 17;9:e76978. doi: 10.2196/76978 (PMC12489404; doi:10.2196/76978)
Supplement: Multimedia Appendix 3 [file formative_v9i1e76978_app3.docx]

## Multimedia Appendix 3: Structured Interview Guide

The following structured-interview questions were used to explore patients’ experiences with the digital lifestyle monitoring system:

|  | **General Cardiac Rehabilitation** |
| --- | --- |
| 1 | How did you experience the year after your hospital treatment in general? |
| 2 | Has your daily life changed since your treatment? |
| 3 | Did you receive follow-up care after your treatment, or participate in cardiac rehabilitation? |
| 4 | Did you change your lifestyle just before or after your treatment? |
| 5 | Did you set specific goals for these lifestyle changes? |
| 6 | Were you able to maintain these lifestyle changes? |
| 7 | What helped you most in sustaining these lifestyle changes? |
| 8 | What made it difficult to maintain these lifestyle changes? |
| 9 | Are there any factors that influence your lifestyle (considering diet, stress, sleep, exercise)? |
|  | **Study in General** |
| 10 | What was your motivation for participating in the study? |
| 11 | Were there any considerations that made you hesitant to participate in the study? If so, what were they? |
| 12 | Can you describe your overall experience with the study? |
| 13 | Did the study fit your personal daily situation? Please explain. |
| 14 | Were you able to follow the study plan effectively (answering the chatbot, wearing the watch, and completing the quarterly questionnaires)? What helped or hindered you in this? |
|  | **NASA Workload – Time Investment/Effort** |
| 15 | How much effort and time did participating in the study require? (*On a paper-scale from 1 to 100 in 5-points steps, where 1 =”very little effort” and 100=”very high effort”*) |
|  | **Lifestyle Awareness and Behavior Change** |
| 16 | What role did your participation in this study play in your care pathway? |
| 17 | To what extent did participation in the study help you gain insight into your lifestyle? |
| 18 | To what extent did participation in the study influence your lifestyle behaviors, and how did it affect your ability to maintain these changes? |
|  | **System Usability** |
| 19 | How do you generally use digital and online tools such as apps or websites? |
| 20 | Have you previously used tools to monitor your lifestyle? |
| 21 | Did you use any other tools to monitor your lifestyle over the past year? |
| 22 | What were your first impressions when you started using the system? |
| 23 | How did you experience the self-monitoring system overall? |
|  | **Specific System Components** |
| 24 | Dashboard |
|  | - Did you use the dashboard? Yes/No |
|  | - If "No": Why not? |
|  | - What was your overall experience with the dashboard? |
|  | - To what extent were the dashboard visualizations clear and insightful? |
|  | - Can we improve the visualizations from your perspective? If so, how? |
| 25 | Health Watch |
|  | - What was your overall experience with the health watch? |
|  | - What was your overall experience with the health watch application? |
|  | - Did you encounter any barriers to wearing the health watch? |
| 26 | Chatbot |
|  | - What was your overall experience with the chatbot? |
|  | - Did you encounter any barriers in answering the chatbot questions? |
|  | - Can we improve the chatbot from your perspective? If so, how? |
| 27 | Quarterly Questionnaires |
|  | - What was your overall experience with the quarterly questionnaires? |
|  | - Did you use the results page? |
|  | - Can we improve this functionality from your perspective? |
| 28 | Goal-Setting Functionality |
|  | - Did you use the goal-setting functionality? Yes/No |
|  | - If "No": Why not? |
|  | - What was your overall experience with this functionality? |
|  | - Can we improve this functionality from your perspective? |
| 29 | Sharing with Relatives |
|  | - Did you use the function to share your data with someone else? Yes/No |
|  | - If "No": Why not? |
|  | - What was your overall experience with this functionality? |
|  | - Can we improve this functionality from your perspective? |
| 30 | Which part of the system were you most enthusiastic about? |
| 31 | From your perspective, is anything missing from the system? |
| 32 | What were your biggest frustrations with the self-monitoring system? |
| 33 | What could make your experience with the system more enjoyable? |
| 34 | Would you like to continue using the system in the future? Please explain. |
| 35 | If the self-monitoring system were integrated in standard health care, what would be the ideal design for you? |
|  | **Conclusion** |
| 36 | Is there anything from this study that you will take with you into the future? |
| 37 | Do you have any additional questions or comments? |
